# Early emergence of *Yersinia pestis* as a severe respiratory pathogen

**DOI:** 10.1038/ncomms8487

**Published:** 2015-06-30

**Authors:** Daniel L. Zimbler, Jay A. Schroeder, Justin L. Eddy, Wyndham W. Lathem

**Affiliations:** 1 Department of Microbiology-Immunology, Northwestern University Feinberg School of Medicine, Chicago, Illinois 60611, USA

## Abstract

*Yersinia pestis* causes the fatal respiratory disease pneumonic plague. *Y. pestis* recently evolved from the gastrointestinal pathogen *Y. pseudotuberculosis*; however, it is not known at what point *Y. pestis* gained the ability to induce a fulminant pneumonia. Here we show that the acquisition of a single gene encoding the protease Pla was sufficient for the most ancestral, deeply rooted strains of *Y. pestis* to cause pneumonic plague, indicating that *Y. pestis* was primed to infect the lungs at a very early stage in its evolution. As *Y. pestis* further evolved, modern strains acquired a single amino-acid modification within Pla that optimizes protease activity. While this modification is unnecessary to cause pneumonic plague, the substitution is instead needed to efficiently induce the invasive infection associated with bubonic plague. These findings indicate that *Y. pestis* was capable of causing pneumonic plague before it evolved to optimally cause invasive infections in mammals.

## Abstract (web summary)

*Yersinia pestis*, which evolved from a gastrointestinal pathogen, causes pneumonic and bubonic plague. Here Zimbler *et al.* show that the gain of a single protein enabled *Y. pestis* to first cause pneumonic plague, and one amino-acid change in the same protein then allowed the bacteria to efficiently cause bubonic plague.

## Graphical abstract

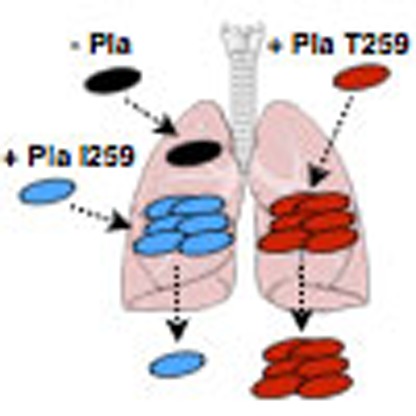

The adaptation of bacterial pathogens to new hosts and specific microenvironments within the body typically occurs via the combined gain and loss of genetic elements during the evolution of the species. *Y. pestis,* the causative agent of plague^1^, represents an exceptional system for understanding how a pathogen adapts to new host environments to cause disease due to its recent emergence as a human pathogen and its relatively clonal nature^2^,^3^,^4^. The divergence of *Y. pestis* from its progenitor species *Y. pseudotuberculosis* occurred within the last 10,000 years through multiple distinct genetic gains and losses, resulting in strikingly different modes of transmission and pathogenicity^2^,^4^,^5^. The soil- and water-borne enteropathogen *Y. pseudotuberculosis* causes the mild, self-limiting disease yersiniosis and is transmitted by the faecal–oral route^1^. In contrast, *Y. pestis* is transmitted by fleabites or aerosols, and causes the severely invasive and virulent disease plague^1^. Investigations on the genetic history of 118 annotated genomes of *Y. pestis* have revealed an evolutionary lineage that has defined both early ancestral (branch 0) and modern pandemic (branches 1 and 2) populations of *Y. pestis*^6^ based on sequential single-nucleotide polymorphism changes (Fig. 1a).

Indeed, recent work has begun to examine and retrace the specific genetic changes that were necessary for the transition from a mild self-limiting enteric disease to a severely invasive and virulent disease^7^,^8^,^9^,^10^,^11^. This change in disease and transmission is attributed to both the acquisition of new chromosomal and extrachromosomal DNA and as well as through genomic decay^9^,^12^. For example, the genes encoding for O-antigen synthesis on its lipopolysaccharide are inactivated in *Y. pestis*, resulting in a rough form of lipopolysaccharide^13^. *Y. pestis* also contains disruptions in the genes for the adhesins invasin and YadA^14^, both of which are functional in *Y. pseudotuberculosis*^15^,^16^. Furthermore, a recent study by Sun *et al.*^7^ has described only four minor genetic changes that were necessary for the flea-borne transmission of *Y. pestis*, which contributed directly to the ability of this pathogen to cause bubonic plague. In addition, the gain of the two plasmids pMT1 and pPCP1 that encode genes contributing directly to survival and virulence in both the flea and mammal is an apparent distinction of *Y. pestis* as it evolved from *Y. pseudotuberculosis*^17^.

*Y. pestis* can cause bubonic, septicemic and pneumonic plague in mammals. Of these, pneumonic plague is a unique respiratory syndrome that results in a purulent, multifocal, severe exudative bronchopneumonia and is the deadliest form of the disease, with fatality rates approaching 100% if untreated^1^,^18^. The disease is characterized as biphasic: the early phase is relatively asymptomatic and non-inflammatory, while the latter phase is highly pro-inflammatory, resulting in massive lobar lesions consisting primarily of infiltrating neutrophils, upregulation of cytokine and chemokine levels, and bacterial dissemination^18^.

One of the major virulence determinants encoded on the pPCP1 plasmid that is required for the development of pneumonic plague is the protease Pla. Similar in sequence and structure to OmpT of *Escherichia coli* and PgtE of *Salmonella enterica*, this β-barrel omptin family aspartic protease has been shown to have a wide range of proteolytic, adhesive and invasive properties necessary for *Y. pestis* pathogenesis in mammals^19^,^20^,^21^. While the most well-studied activity of Pla is the cleavage of host plasminogen (Plg) into the activated serine protease plasmin, to date only the host apoptotic molecule Fas ligand (FasL) has been defined *in vivo* as a substrate of Pla that contributes to primary pneumonic plague^22^,^23^,^24^. The amino-acid sequence of Pla is 100% conserved among all modern branched strains of *Y. pestis*; however, comparative genomics and substitutional analyses indicate that the sequence of Pla has shifted since the gene was introduced into ancestral branched strains of *Y. pestis* (Fig. 1a)^8^,^25^. In ancestral lineages such as Angola and Pestoides, Pla contains a single isoleucine substitution at position 259 (I259) located on the surface loop 5, whereas the modern lineages KIM and CO92 have a conserved threonine at this residue (T259)^26^,^27^. This single change has been shown *in vitro* to increase the efficiency of Plg activation and plasmin stability formed by the T259 variant of Pla^28^.

The acquisition of pPCP1/Pla is positioned as an early occurrence in the emergence of pandemic strains of *Y. pestis*; however, there still exist ancestral isolates, such as Pestoides E and F (isolated from voles in the Transcaucasian highland)^29^, that represent an intermediate lineage between *Y. pseudotuberculosis* and modern *Y. pestis* and do not carry pPCP1. However, these naturally occurring isolates of *Y. pestis* that do not harbour pPCP1 have nevertheless been reported to both colonize fleas and infect rodents^30^,^31^,^32^. Moreover, DNA of the pPCP1 plasmid, containing the modern T259 variant of Pla, has been detected in the remains of victims from both the Justinian plague and the Black Death, implying that most pandemic strains contained the genetic potential to be as virulent as strains currently associated with human disease^33^,^34^.

This study was undertaken to identify and examine when during its emergence *Y. pestis* gained the ability to cause severe primary pneumonic plague. By utilizing the deeply rooted, ancestral Pestoides and Angola lineages, we demonstrate that the specific microevolutionary event of pPCP1/Pla acquisition contributed directly to the adaptation of *Y. pestis* to the respiratory environment, and no additional genetic changes were necessary to cause pneumonic plague. On the other hand, our results indicate that the T259 modification of the Pla protease enhanced systemic disease, suggesting that *Y. pestis* was primed to cause a fulminant pneumonia before its ability to efficiently cause invasive infections. Finally, we propose that the I259T substitution of Pla may have been one of the defining events that enabled pandemic spread of *Y. pestis.*

## Results

### Ancestral strains of *Y. pestis* rapidly outgrow in the lungs of mice

**Figure 1:**
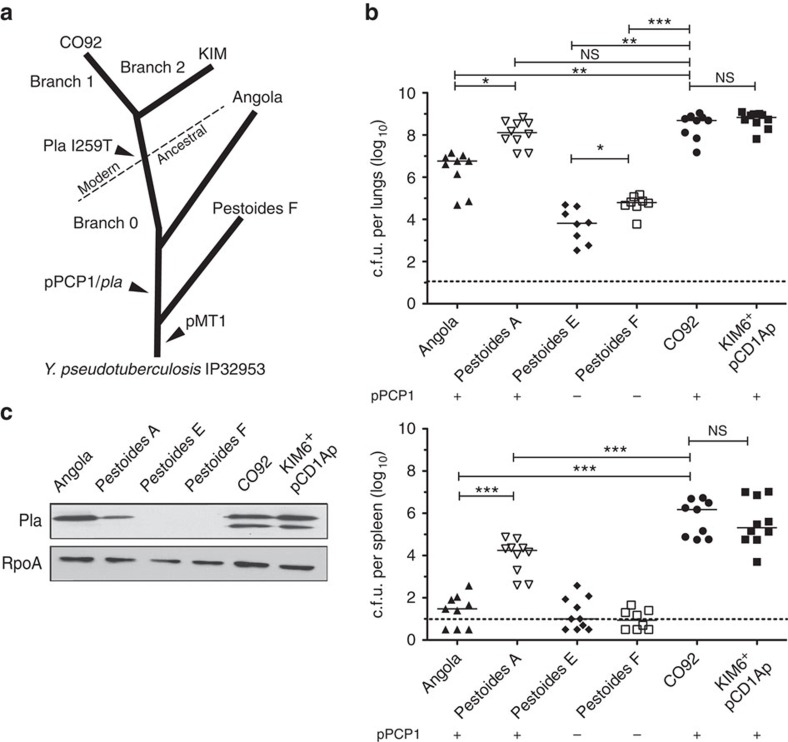
pPCP1 is required by ancestral *Y. pestis* to cause primary pneumonic plague. (**a**) Genomic maximum parsimony tree and divergence based on 16 *Y. pestis* genomes. The division between modern, pandemic strains and ancestral isolates is indicated. Tree was adapted from Morelli *et al*.^5^ (**b**) Bacterial burden within the lungs and spleens of mice (*n*=10) infected i.n. with the indicated *Y. pestis* strains. Each point represents the numbers of bacteria recovered from a single mouse at 48 h post inoculation. The limit of detection is indicated by the dashed line and symbols in the dotted line indicate c.f.u. below the limit of detection. Symbols below the limit of detection represent mice that did not have detectable numbers of bacteria. A solid line indicates the median of c.f.u. recovered. The presence or absence of pPCP1 in each strain is indicated below. (**c**) Immunoblot analysis of whole-cell lysates of the indicated *Y. pestis* strains with antibodies to Pla and RpoA (as a loading control). The lower band represents the autoprocessed form of Pla (see Supplementary Fig. 5a). Full blots are shown in Supplementary Fig. 6. Panel is representative of three independent replicates. Data are combined from two independent experiments and error bars represent the s.e.m. (*P≤0.05, **P≤0.01, ***P≤0.001, NS, not significant by Mann–Whitney *U*-test).

It is not known as to when during its emergence from the gastrointestinal pathogen *Y. pseudotuberculosis* that ancestral *Y. pestis* gained the ability to cause severe primary pneumonic plague. To address this question, we examined the capacity of the deeply rooted, ancestral *Y. pestis* strains Angola, Pestoides A, E and F (branch 0) (Fig. 1a), which display biochemical phenotypes and have environmental niches that are distinct from modern pandemic lineages and have not been associated with human disease^31^,^35^,^36^, to establish an infection in the lungs. We compared the bacterial outgrowth of these strains to that of the well-studied, modern-positioned lineage strains CO92 and KIM (branches 1 and 2) (Fig. 1a), both isolated from human plague cases, in an intranasal (i.n.) mouse model of pneumonic plague. We found that both Angola and Pestoides A, ancestral branched strains that carry pPCP1 (although with the I259 variant of Pla), are able to replicate to high levels in the lungs, approaching or equivalent to the bacterial burdens of the modern lineage strains CO92 and KIM (Fig. 1b). On the other hand, the Pestoides E and F strains are unable to rapidly proliferate in the pulmonary compartment (Fig. 1b), suggesting the absence of one or more virulence determinants required to cause primary pneumonic plague.

A comparison of these strains showed that a major distinguishing feature between Angola and Pestoides A versus Pestoides E and F is the presence of pPCP1, the plasmid encoding the virulence factor Pla; neither Pestoides E nor Pestoides F naturally carry pPCP1 (Supplementary Fig. 1a)^29^. Consistent with this, we found that all of the pPCP1^+^ strains are able to synthesize Pla to similar levels, however both Angola and Pestoides A do not autoprocess Pla due to the I259 variant of the protease carried by these strains^28^ (Fig. 1c). Thus, these data suggest that the gain of pPCP1 early during the evolution of *Y. pestis* was necessary for rapid bacterial outgrowth in the lungs.

### Pestoides F is unable to cause primary pneumonic plague

**Figure 2:**
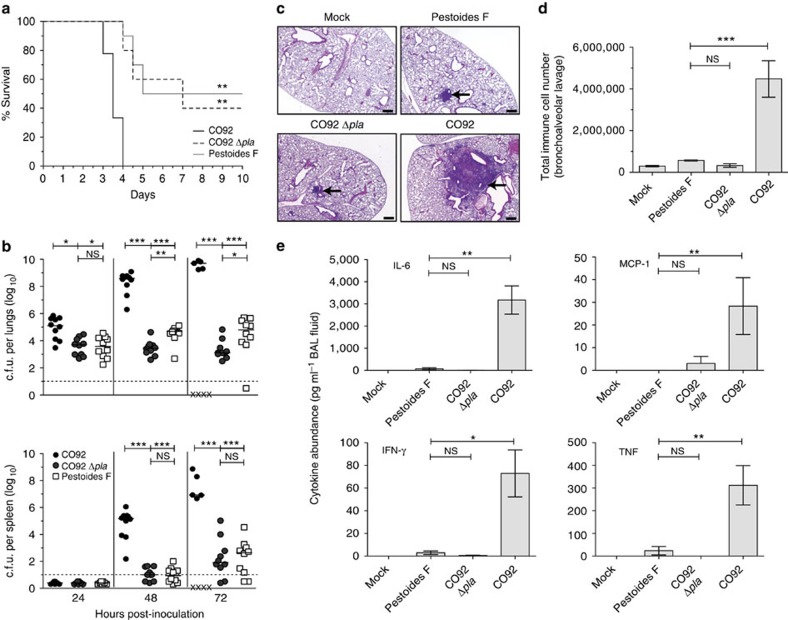
Pestoides F resembles Δ*pla Y. pestis* during intranasal infections. (**a**) Survival of mice (*n*=20) infected i.n. with *Y. pestis* CO92, CO92 Δ*pla* or Pestoides F. (**b**) Bacterial burden within the lungs and spleens of mice (*n*=10) infected i.n. with the indicated *Y. pestis* strains, as described in Fig. 1. By 72 h, mice begin to succumb to the CO92 infection (indicated by “X” on the *x* axis). (**c**) Pathology of mouse lung sections stained with H&E at 48 h post inoculation with PBS (mock), Pestoides F, CO92 Δ*pla* or CO92 *Y. pestis*. Representative images of inflammatory lesions are shown (arrows; *n*=3). Scale bar, 200 μm. (**d**) Enumeration of total immune cells present in BAL fluid 48 h post inoculation with PBS (mock), Pestoides F, CO92 Δ*pla* or CO92 *Y. pestis* (*n*=10). (**e**) Abundance of the indicated inflammatory cytokines present in BAL fluid at 48 h post inoculation. Data are combined from two independent experiments and error bars represent the s.e.m. (*P≤0.05, **P≤0.01, NS, not significant by Log-Rank test (survival), Mann–Whitney *U*-test (c.f.u.)). H&E, haematoxylin/eosin.

As Pestoides F is considered to be one of the most ancestral existing isolates of *Y. pestis*^6^,^35^ after it emerged from *Y. pseudotuberculosis* but before the acquisition of pPCP1, we further examined the interaction of this strain with the respiratory environment and in comparison with a mutant of CO92 lacking *pla*. To confirm that Pestoides F does not display functional activity generally attributed to Pla, we validated that Pestoides F is unable to activate Plg, similarly to an isogenic Δ*pla* mutant of CO92 *Y. pestis* (Supplementary Fig. 1b,c). We then infected mice via the i.n. route and followed survival over time; while all mice infected with the wild-type CO92 strain rapidly succumbed to the infection by day 4 post infection, the Pestoides F*-*infected mice showed a survival curve similar to that of Δ*pla* CO92 and were significantly delayed in time to death compared with CO92 (Fig. 2a). To further assess the kinetics of infection of Pestoides F compared with CO92, we enumerated the colony-forming unit (c.f.u.) in the lungs and spleens at various times post infection and found that neither the Δ*pla* CO92 mutant nor the ancestral Pestoides F isolate are unable to replicate to high levels in the pulmonary compartment as compared with modern CO92 (Fig. 2b). Interestingly, by 48 h Pestoides F is able to modestly but significantly outgrow by ∼10–100-fold in the lungs compared with mice infected with the Δ*pla* CO92 mutant, although similar numbers of bacteria are detected in spleens of Pestoides F and Δ*pla* CO92-infected mice at all time points examined (Fig. 2b).

To test the impact of Pestoides F on the host response during lung infection, we first examined the pathology of mouse lungs at 48 h post infection. Mice infected with wild-type CO92 produced large lobar pulmonary lesions throughout the lungs, whereas the lungs of Pestoides F-infected mice showed smaller, more nodular inflammatory lesions, similar in size and distribution to that of the Δ*pla* CO92 (Fig. 2c). We then performed flow cytometry to quantitate the observed influx of infiltrating cells into the lung airspace and to determine the distribution of cell types within the total cell population. CO92-infected mice showed a massive increase in the total number of cells recovered by bronchoalveolar lavage (BAL), and consistent with earlier reports^37^,^38^ the primary infiltrating cell type recruited to the lungs is the neutrophil (Fig. 2d; Supplementary Fig. 2). In contrast, mice infected with Pestoides F or the isogenic Δ*pla* CO92 mutant do not respond with this influx of immune cells to the lungs (Fig. 2d; Supplementary Fig. 2). Furthermore, an examination of the pulmonary inflammatory cytokine response reveals that, unlike CO92, which stimulates a robust release of multiple inflammatory cytokines into the airspace, mice infected with Pestoides F or the Δ*pla* CO92 mutant exhibit a severely dampened response with significantly lower cytokine levels (Fig. 2e). Thus, our data demonstrate that the ancestral Pestoides F strain is unable to cause primary pneumonic plague in a manner that is highly similar to a Δ*pla* mutant of CO92.

### Pestoides F is competent to produce active Pla

**Figure 3:**
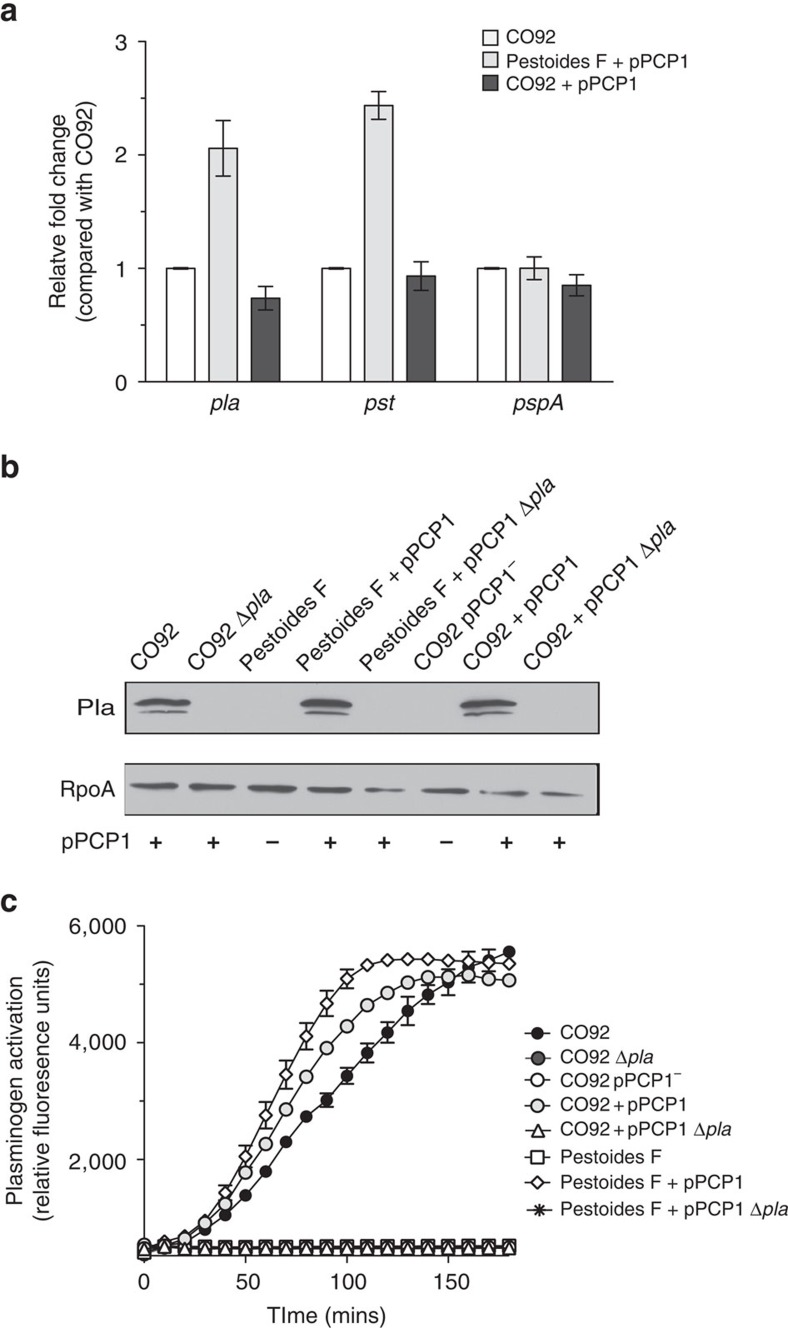
Pestoides F is competent to carry pPCP1 and produce active Pla. (**a**) Relative copy number of pPCP1 (represented by the pPCP1-encoded genes *pla* and *pst*) in the pPCP1-reintroduced Pestoides F and CO92 strains, compared with CO92 (set at 1). *pspA* was used as a control for a chromosomal gene. Relative copy number for each gene was measured by quantitative PCR from gDNA isolated from cultures grown overnight at 37 °C and normalized to *gyrB*. Data are combined from three independent biological replicates repeated twice; error bars represent the s.e.m. (**b**) Immunoblot analysis of whole-cell lysates of indicated *Y. pestis* strains grown at 37 °C with antibodies against Pla and RpoA (as a loading control). Panel is representative of three independent replicates. The presence or absence of pPCP1 in each strain is indicated. Full blots are shown in Supplementary Fig. 6. (**c**) The Plg-activating ability of the indicated *Y. pestis* CO92 or Pestoides F strains cultured at 37 °C is shown. Data are representative of three independent experiments performed in triplicate; error bars represent the s.e.m.

On the basis of the similarities of the respiratory infection between Pestoides F and CO92 Δ*pla*, we asked whether the acquisition of pPCP1 (and specifically Pla) was sufficient for this ancestral *Y. pestis* strain to cause primary pneumonic plague. However, it is not yet known whether Pestoides F is able to stably harbour pPCP1 and produce active Pla. To test this, we introduced a kanamycin-marked pPCP1 derived from CO92 (ref. 14) into the pCD1^+^ and pCD1^−^ strains of Pestoides F. To ensure that this marked version of pPCP1 did not affect its encoded functions, we reintroduced the kanamycin-marked pPCP1 plasmid back into the pCD1^+^ and pCD1^−^ strains of CO92 lacking pPCP1; in all cases, the kanamycin resistance marker was subsequently excised by Flp-based recombination. To test the ability of Pestoides F to carry pPCP1, we measured the copy number of the *pla* and *pst* genes (both encoded on pPCP1) in Pestoides F relative to that in CO92 and Δ*pla* CO92 carrying the reintroduced pPCP1. The copy number of pPCP1 carried by wild-type CO92 and the unmarked, reintroduced pPCP1 in CO92 are similar, indicating that the scar left by removal of the antibiotic cassette has no effect on plasmid replication (Fig. 3a). In addition, we found that Pestoides F naturally maintains pPCP1 without antibiotic selection (albeit at a higher relative copy number compared with CO92; Fig. 3a). Furthermore, neither Angola nor Pestoides A maintain pPCP1 at a significantly different copy number compared with wild-type CO92 (Supplementary Fig. 3a). To determine the conservation of *pla* regulation between ancestral and modern strains, a P*pla*-gfp reporter containing the CO92 *pla* promoter cloned upstream of the coding sequence (CDS) for the green fluorescent protein (GFP) was integrated onto the chromosomes of CO92, Angola, Pestoides A and Pestoides F at the *att*Tn7 site. Bacteria were cultured at 37 °C and the fluorescence of each strain was measured and normalized to the optical densities of the cultures. No significant change in fluorescence was observed between any of the ancestral strains compared with that of CO92 (Supplementary Fig. 3b), suggesting no differences in the regulation of *pla* transcription within these strains. Finally, immunoblot analysis of these strains cultured under the same conditions demonstrates that Pestoides F is able to synthesize Pla from pPCP1 at a similar level to that of CO92 (Fig. 3b).

To determine whether Pla properly localizes to the outer membrane and exhibits enzymatic activity when expressed in Pestoides F, we conducted a Plg activation assay and found that Pestoides F carrying pPCP1 showed similar levels of Plg activation compared with CO92 (Fig. 3c). Furthermore, additional Pla activity of Pestoides F was demonstrated by the similar efficiency as CO92 in the degradation of FasL (Supplementary Fig. 3c). The Pla activity of these Pestoides F or CO92 strains was subsequently eliminated in the isogenic Δ*pla* mutants (Fig. 3c; Supplementary Fig. 3c). Together, these data show that the ancestral strain Pestoides F can harbour pPCP1 and produce proteolytically active Pla, indicating that additional changes to the *Y. pestis* genome were not necessary for the expression, production and activity of this essential virulence factor.

### Pla is sufficient for Pestoides F to cause pneumonic plague

**Figure 4:**
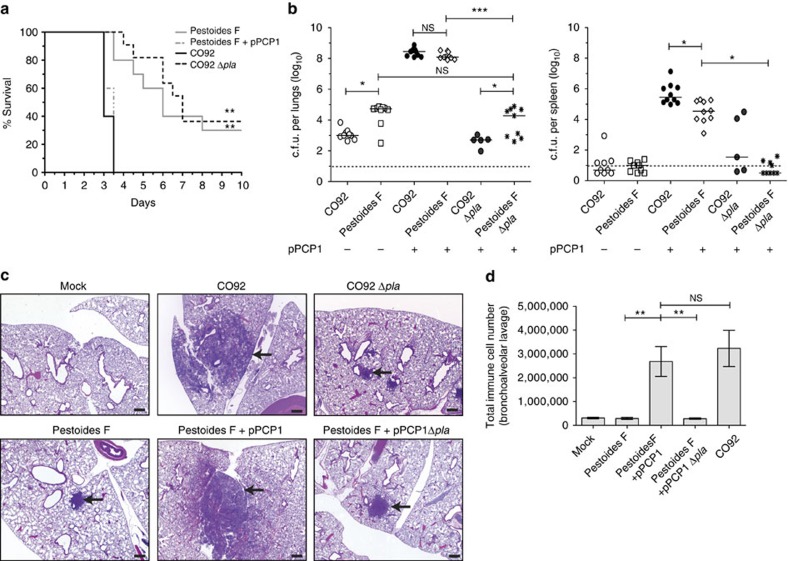
The acquisition of Pla by Pestoides F is sufficient to cause primary pneumonic plague. (**a**) Survival of mice (*n*=10) infected i.n. with the indicated strains of *Y. pestis.* (**b**) Bacterial burden within the lungs and spleens of mice (*n*=10) infected i.n. with the indicated Pestoides F or CO92 strains, as described in Fig. 1. The presence or absence of pPCP1 is indicated. (**c**) Pathology of mouse lung sections stained with H&E at 48 h post inoculation with PBS (mock) or the indicated *Y. pestis* strains. Representative images of inflammatory lesions (arrows; *n*=3) are shown. Scale bar, 200 μm. (**d**) Enumeration of total immune cells present in BAL fluid 48 h post inoculation with PBS (mock) or the indicated *Y. pestis* strains (*n*=10). Data are combined from two independent experiments. Error bars represent the s.e.m. (*P≤0.05, **P≤0.01, ***P≤0.001, NS, not significant; Log-Rank test (survival), Mann–Whitney *U*-test (c.f.u.)). H&E, haematoxylin/eosin.

Pla is necessary for the progression of pneumonic plague in modern lineages of *Y. pestis*; therefore, we tested whether the acquisition of Pla by Pestoides F was all that was required for one of the earliest existing ancestral strains of *Y. pestis* to cause primary pneumonic plague. On introduction to mice via the i.n. route, we found that Pestoides F carrying pPCP1 was able to grow to high levels in the lungs and cause the death of the animals within 3–3.5 days, to the same extent and rate as wild-type *Y. pestis* CO92 (not statistically different, Mann–Whitney *U*-test; Fig. 4a,b). This increase in bacterial burden within the lungs is Pla dependent in both Pestoides F and CO92, as the time to death and enumerated c.f.u. were significantly decreased in the Δ*pla* isogenic mutant infections in both backgrounds (Fig. 4a,b). An examination of the host response to Pestoides F harbouring pPCP1 revealed large lobar pulmonary lesions (Fig. 4c), a significant increase in total cell number (Fig. 4d) with neutrophils being the primary infiltrating cell type (Supplementary Fig. 4a), and a robust inflammatory cytokine response, all which are similar to that caused by modern *Y. pestis* strain CO92 (Supplementary Fig. 4b). In total, our results demonstrate that the acquisition pPCP1/Pla by one of the most deeply rooted strains of *Y. pestis* was sufficient for this newly emerged species to cause primary pneumonic plague within rodents.

### Pla I259T modification was not required to cause pneumonic plague

A single residue substitution (I259T) in Pla between ancestral and modern lineages of *Y. pestis* (Fig. 1a) indicates that Pla has undergone natural adaptation since its acquisition by *Yersinia*. Therefore, we asked whether this modification was required by or enhanced the ability of *Y. pestis* to cause primary pneumonic plague. To do so, we introduced the I259 amino-acid substitution into Pestoides F+pPCP1 as well as into the modern CO92 isolate, thus generating the ancestral variant of Pla within both ancestral and modern *Y. pestis* strains. Immunoblot analysis of these strains confirms that the I259 variant of Pla does not autoprocess itself and has a reduced rate of Plg activation compared with the T259 variant in both modern and ancestral *Y. pestis*, consistent with a previous study on the I259 variant^28^ (Supplementary Fig. 5a,b). However, the I259 Pla variant is still able to degrade FasL to the same efficiency as Pla T259 (Supplementary Fig. 5c,d). We also examined the rate of Plg activation by Pestoides A (which expresses the I259 variant of Pla), and observed that this isolate has reduced activity even compared with other strains expressing the same I259 variant of Pla (Supplementary Fig. 5b).

**Figure 5:**
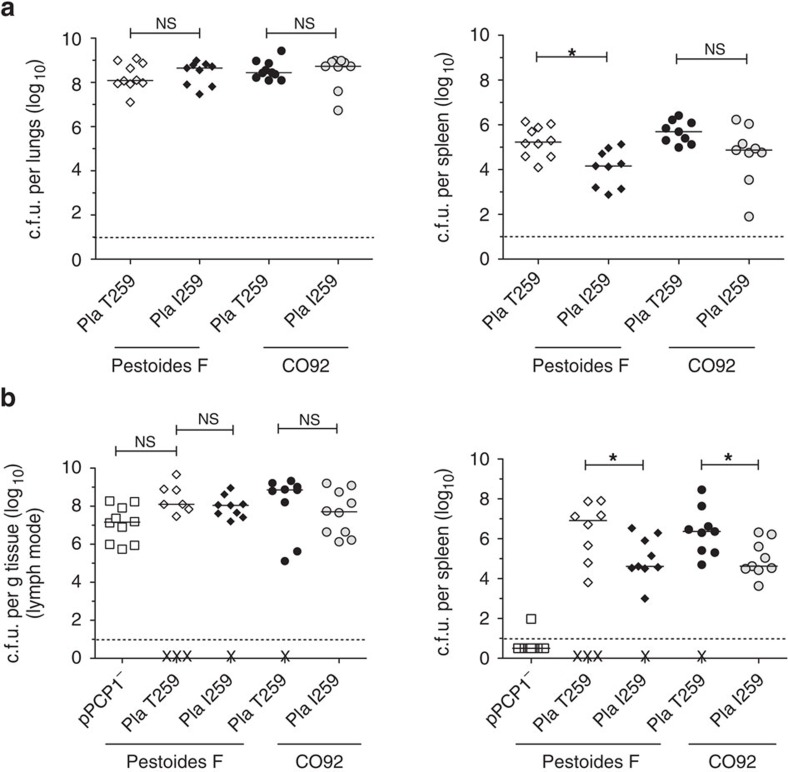
The ancestral variant of Pla is sufficient to cause pneumonic plague but not optimal systemic infection. (**a**) Bacterial burden within the lungs and spleens of mice (*n*=10) infected i.n. with *Y. pestis* CO92 or Pestoides F carrying pPCP1 with either the T259 or I259 variant of Pla, as described in Fig. 1. (**b**) Bacterial burden within the inguinal lymph nodes and spleens of s.c. infected mice (*n*=10) with wild-type Pestoides F, or CO92 or Pestoides F carrying pPCP1 with either the T259 or I259 variant of Pla. The variant of Pla in each strain is indicated. Data are combined from two independent experiments and error bars represent the s.e.m. (*P≤0.05, NS, not significant by Mann–Whitney *U*-test).

We next asked whether the T259 variant of Pla enhances virulence during primary pneumonic plague compared with the Pla I259 in either the modern (CO92) or ancestral (Pestoides F) strains of *Y. pestis* by assessing the bacterial burden in the lungs after 48 h. We found no significant difference (Mann–Whitney *U*-test) in c.f.u. in the lungs of Pestoides F or CO92 expressing the I259 variant of Pla compared with the same strains expressing Pla T259 (Fig. 5a), indicating that this single amino-acid substitution was not required for *Y. pestis* to infect the lungs and rapidly replicate. However, there were significantly fewer bacteria in the spleens of mice infected with Pla I259 compared with the T259 variant in the Pestoides F isolate (Fig. 5a). Together with the data presented in Fig. 4, our results indicate that once *Y. pestis* acquired Pla, no additional genetic changes were required for the plague bacillus to cause a rapidly progressing pneumonic infection; rather this modification may instead be involved in dissemination from the lungs and/or bacterial survival during systemic infection.

### Pla I259T modification was necessary for invasive infections

As we observed reduced numbers of bacteria in the spleens following i.n. infection with Pestoides F+Pla I259 compared with Pla T259, the data shown in Fig. 5a led us to hypothesize that this modification of Pla may be more significant for dissemination or survival of *Y. pestis* during systemic, invasive infections, such as occurs during bubonic plague. To test this, we used the same strains in a subcutaneous (s.c.) mouse model of infection of bubonic plague and measured bacterial burden within the inguinal lymph nodes and spleens of infected mice. Mice infected with either the ancestral or modern Pla variant in the Pestoides F or CO92 background resulted in equivalent bacterial outgrowth within the inguinal lymph node after 3 days (Fig. 5b). However, we detected ∼100-fold fewer bacteria in the spleens of mice infected with the ancestral I259 variant of Pla compared with Pla T259 in both the ancestral and modern strains of *Y. pestis* (Fig. 5b). Moreover, Pestoides F is able to outgrow in the lymph node; however, it only disseminates to distal sites by day 3 when harbouring pPCP1 (Fig. 5b). Thus, these observations indicate that the I259T modification of Pla was important for *Y*. *pestis* to cause a disseminated, invasive infection from the site of inoculation, such as occurs during bubonic plague, in both modern and ancestral strains of *Y. pestis*.

## Discussion

The mechanisms by which new bacterial pathogens emerge are not well understood, particularly in regards to the specific gain or loss of individual genetic determinants that enable the adaptation and development of acute disease in discrete hosts and/or microenvironments. These genetic changes are believed to account for phenotypic distinctions and pathogenic potential observed between lineages of *Y. pestis*, as genetic diversity is a known source of phenotypic diversity underlying disease dynamics.^39^,^40^ However, the molecular mechanisms that *Y. pestis* acquired to specifically become a severe respiratory pathogen are not yet established, despite several comparative genomic, transcriptomic and proteomic studies^6^,^8^,^41^.

Before this study, it was unknown whether ancestral strains of *Y. pestis* were capable of causing primary pneumonic plague and at what point during the evolution of *Y. pestis* they became competent to do so. Here we report that ancestral, branch 0 strains of *Y. pestis* gained the ability to successfully replicate to high levels within the pulmonary compartment very early in their evolution from *Y. pseudotuberculosis*, but only if they had acquired the gene encoding the Pla protease. This provides additional support for the acquisition of pPCP1 as a critical step in the adaptation of *Y. pestis* to the pulmonary environment. While it has been demonstrated that *Y. pseudotuberculosis* can colonize the lungs and stimulate a pneumonia in mice, the disease is delayed and does not exhibit the pulmonary pathology or rapid systemic spread that is observed during infections with *Y. pestis*^42^. Furthermore, *Y. pseudotuberculosis* is not known to naturally cause epidemics of respiratory disease.

While Pestoides F is one of the earliest existing *Y. pestis* strains in branch 0 following the divergence of the species from *Y. pseudotuberculosis* (Fig. 1a), here the addition of pPCP1 now confers Pestoides F with the higher pathogenicity observed in respiratory infections that are exhibited by the modern CO92 and KIM strains. This is the only genetic change needed by one of the most deeply rooted of all *Y. pestis* strains to cause a fulminant lung infection, and indicates that even during the transitional evolutionary state between *Y. pseudotuberculosis* and modern *Y. pestis*, this ancestral species was primed to cause pneumonic plague. In contrast, the addition of pPCP1 to *Y. pseudotuberculosis* does not result in proteolytically active Pla or enhance virulence in mice, as Pla produced by *Y. pseudotuberculosis* is sterically inhibited by the presence of O-antigen on the bacterial surface^24^,^43^,^44^. While the pPCP1 plasmid itself is not sufficient to explain all of the differences in virulence between *Y. pseudotuberculosis* and *Y. pestis*, our data does indicate that ancestral strains could initiate epidemics of pneumonic plague as soon as pPCP1 was acquired.

The origin of the pPCP1 plasmid remains unknown, although it encodes a ColE1-like origin of replication, suggesting a common ancestry with plasmids typically found in enteric organisms^31^. The plasmid encodes three proteins; thus far Pla is the only virulence determinant encoded on pPCP1 shown to be important for development of enhanced disease by modern *Y. pestis*^37^,^45^, a conclusion that is further supported by this work. In addition, our data clearly indicate that not only the acquisition but also the modification of Pla was a crucial adaptation in the systemic spread of *Y. pestis* from both the lungs and the lymph node. The prominent function of Pla is thought to be the proteolytic activation of host Plg into plasmin, which allows the bacteria to escape from immobilizing fibrin clots and potentiates fibrinolysis. During infection with *Y. pestis* carrying the I259 variant of Pla, colonization and outgrowth within the lungs are unaffected; however, dissemination is significantly reduced compared with the T259 variant, likely due to reduced Plg activation and stable plasmin formation by Pla during infection. During pneumonic plague, mortality is due to the pneumonia developed within the lungs, whereas during bubonic plague the animal dies from sepsis due to dissemination of bacteria from the lymph node, and not from localized lymphadenopathy^17^,^18^,^22^. As our data show that the I259T substitution significantly enhanced the invasive capacity of *Y. pestis* during bubonic plague, it is tempting to speculate that this modification of Pla may have contributed to the ‘big bang' event, recently proposed by Cui *et al.*^6^, that resulted in the transition from localized outbreaks of plague to the pandemic spread of *Y. pestis*.

This study expands on previous work by Welkos *et al.*^46^ and Worsham and Roy^30^, which showed that Pestoides F has a comparable LD_50_ (dose lethal to 50%) to CO92 in both the aerosol and s.c. routes of infection. Data presented here suggest that the outcomes of disease observed in these earlier reports are likely due to bacteremia following escape of Pestoides F from the lungs or via direct inoculation into the blood following s.c. or intradermal injection (such as observed with *Y. pestis* CO92 Δ*pla*),^17^,^37^ rather than the severe exudative bronchopneumonia or bubonic infection that occurs with pPCP1^+^ strains. Indeed, Pla is dispensable once *Y. pestis* enters the blood^45^, suggesting that pPCP1^−^ strains such as Pestoides E and F may be maintained in the environment at low levels through direct bloodstream infections via flea or animal bite. Furthermore, in light of these previous studies, our data suggest that the acquisition of Pla enabled new, more rapidly progressing forms of disease caused by *Y. pestis*, rather than increased lethality.

The ability of Pestoides F to modestly outgrow in the pulmonary compartment compared with Δ*pla* CO92 supports its placement as a transitional lineage of *Y. pestis*; however, it is unable to cause pneumonic plague. This observation can best be explained by Pestoides F maintaining specific variations in metabolic pathways that allow for increased fitness within the host. Comparative genomics and transcriptomics have revealed frameshifts, point mutations and pseudogenes, which may account for phenotypic distinction in nutritional requirements, carbohydrate fermentation and other biochemical properties between CO92 and Pestoides F^35^,^39^,^41^. For instance, the ability to catabolize L-aspartate into fumarate by aspartase (AspA) has been lost by modern *Y. pestis* lineages such as CO92 and KIM, whereas *Y. pseudotuberculosis* and the ancestral Angola and Pestoides isolates of *Y. pestis* maintain AspA activity^47^. On the other hand, although the Angola isolate encodes *pla*, this strain is not able to grow to the same levels in the lungs as modern isolates, suggesting that Angola may have diverged from the branch 0 group in a way that has ultimately reduced its fitness in the pulmonary compartment. It is of interest to determine the specific genetic differences between Angola, the Pestoides and modern *Y. pestis* that could account for these variations in virulence.

Retracing the evolution of *Y. pestis* since its emergence has revealed multiple small genetic changes that have altered host tropism, transmission and virulence^12^,^48^. For example, investigations into the adaptation of *Y. pestis* to flea-borne transmission have uncovered only a few genetic alterations that facilitated this ability^7^,^11^,^49^,^50^. The increasing genomic comparisons of global *Y. pestis* isolates have revealed that it has evolved clonally with little genetic diversity; however, it is becoming apparent that these small genetic mutations resulting in the change of its transmission have been strongly positively selected for during its evolution. The emergence of new pathogenic clones has been directly linked with both the gain and loss of genetic elements by bacteria that were previously thought to be non-virulent^51^. Moreover, single gene acquisition events have been demonstrated to have fundamental effects on an organism's evolutionary adaptation to new hosts^52^,^53^,^54^. Indeed, our data suggest that the emergence of other new respiratory pathogens could occur similarly via relatively small gene acquisition events. Taken together, our results indicate that *Y. pestis* was primed to cause an acute respiratory disease early on during its evolution from *Y. pseudotuberculosis* and that this occurred before *Y. pestis* was able to develop a fully optimized invasive infection.

## Materials and methods

### Reagents, bacterial strains and culture conditions

All reagents used in this work were purchased from Sigma-Aldrich or VWR unless otherwise stated. The bacterial strains used in this work are listed in Supplementary Table 1, plasmids are listed in Supplementary Table 2 and oligonucleotide sequences are given in Supplementary Table 3. Brain–heart infusion (BHI) broth or agar (Difco) was used to maintain *Y. pestis* strains and derivatives. Luria-Bertani broth or agar was used to maintain all *E. coli* strains. Ampicillin (100 μg ml^−1^) or kanamycin (50 μg ml^−1^) was added to the medium as needed. For animal infections, *Y. pestis* strains were cultured in 10 ml BHI in 125 ml Erlenmeyer flasks with shaking at 250 r.p.m. at either 37 °C with the addition of 2.5 mM CaCl_2_ for i.n. infections or 26 °C for s.c. infections^37^,^55^. All experiments using select agent strains of *Y. pestis* were conducted in an approved BSL-3/ABSL-3 facility at Northwestern University.

### Mutagenesis and generation of *Y. pestis* strains

Recombinant DNA procedures were approved by the Institutional Biosafety Committee of Northwestern University. pCD1^+^ was cured from *Y*. *pestis* strains by passage on BHI plates containing magnesium oxalate and incubation at 37 °C^56^. The loss of pCD1 in these strains was further confirmed by PCR and plasmid profile analysis^57^*. Y. pestis* KIM6^+^ was transformed with pCD1Ap by electroporation. Transformants were selected on BHI+ampicillin+Congo Red following growth at 26 °C for 2 days, and transformants were additionally screened for their ability to grow on BHI at 26 °C but were unable to grow on BHI plates containing magnesium oxalate at 37 °C. The presence of pCD1Ap was further confirmed by PCR.

The kanamycin-marked version pPCP1 derived from CO92 was electroporated into pCD1^+^ or pCD1^−^ strains of Pestoides F. To ensure that this variant of pPCP1 remained isogenic with CO92 and did not impact functions associated with pPCP1 (for example, Pla activity), we reintroduced the kanamycin-marked pPCP1 plasmid back into the pCD1^+^ and pCD1^−^ strains of CO92 lacking pPCP1. Tranformants were selected on BHI+kanamycin following growth at 26 °C for 2 days. The kanamycin resistance cassette used for the selection of transformants was excised using Flp recombinase methods by electroporation of pSkippy as described previously^58^. The presence of unmarked pPCP1 in each strain was confirmed by PCR and plasmid profile analysis^57^. Unmarked isogenic derivatives of *pla* were generated in the pCD1^+^ or pCD1^−^ strains introduced with pPCP1 using a modified form of lambda red recombination^37^, and the kanamycin resistance cassette excised as above. The ancestral Pla variant (I259) in the CO92 and Pestoides F strains were generated by the PCR-based method of overlap extension before introduction into the respective strain carrying pWL204 (ref. 37).

### Plg activation assay, cleavage of FasL and immunoblot analyses

The Plg-activating ability of *Y. pestis* strains was assessed^37^,^59^. Briefly, strains were cultured for 6 h at 37 °C before being diluted to 4 × 10^6^ c.f.u. in 20 mM HEPES buffer and combined with purified human glu-Plg (Haematologic Technologies; 4 μg) and the chromogenic substrate D-AFK-ANSNH-iC_4_H_9_-2HBr (SN5; Haematologic Technologies; 50 μM) in a total volume of 200 μl 20 mM HEPES buffer and incubated at 37 °C for 3 h. The absorbance at 460 nm was measured every 10–11 min in a Molecular Devices SpectraMax M5 fluorescence microplate reader. Data are representative of at least three independent experiments.

The cleavage of the Pla substrate FasL by *Y. pestis* strains was assessed as described previously^38^. Briefly, strains were cultured for 6 h at 37 °C before being diluted to 4 × 10^6^ c.f.u. in 20 mM HEPES and combined with recombinant HA-tagged mouse FasL (R&D systems; 0.1 μg) and incubated at 37 °C. Following incubation for 10, 15, 30 or 60 min, bacteria were removed by centrifugation and proteins contained within the supernatant were mixed with reducing sample buffer, separated by SDS–polyacrylamide gel electrophoresis and transferred to nitrocellulose for analysis by immunoblotting with an anti-HA antibody (1:10,000 dilution; Roche, clone 3F10).

To examine Pla production, bacteria were cultured for 6 h at 37 °C and one optical density unit (equivalent to ∼1 × 10^9^ c.f.u.) of each culture was harvested and mixed with reducing sample buffer, separated by SDS–polyacrylamide gel electrophoresis and transferred to nitrocellulose for analysis by immunoblotting with antibodies to Pla (1:200 dilution) or RpoA as a loading control (1:100,000 dilution; Melanie Marketon, Indiana University), as described previously^60^.

### Plasmid copy number determination

The relative copy number of pPCP1 maintained by *Y. pestis* strains was determined as described^61^. Briefly, genomic DNA (gDNA) was isolated using the Wizard gDNA isolation kit (Promega) from bacteria cultured at 37 °C according to the manufacturer's instructions. Quantitative PCR was performed on gDNA with primers specific for *gyrB*, *pspA*, *pst* and *pla* (Supplementary Table 2). Relative plasmid copy number was determined by the formula 2^−ΔCT^, where ΔCT is the threshold cycle difference between *gyrB* and the gene of interest. Statistical differences were determined by the Student's *t*-test from three independent experiments.

### GFP assays

To assess *pla* expression in *Y. pestis* strains, a reporter construct containing 500 base pairs upstream of the *pla* CDS was fused to the CDS of GFP (P_*pla*_-gfp) and integrated onto the chromosome of the indicated *Y. pestis* (pCD1^−^) strains at the *att*Tn*7* site contained within the *glmS*-*gstS* intergenic region via the Tn*7* site-specific transposon^58^,^60^,^62^. Integrations were confirmed by PCR. Strains carrying the P_*pla*_-gfp reporter were assessed for relative fluorescence at 37 °C in triplicate as described previously using a Tecan Safire microplate reader^58^. Background fluorescence subtraction and normalization were performed as described^58^. Statistical differences were determined by the Student's *t*-test from three independent experiments.

### Animal infections

All procedures involving animals were carried out in compliance with protocols approved by the Institutional Animal Care and Use Committee of Northwestern University. C57BL/6 female mice, aged 6–8 weeks, were obtained from The Jackson Laboratories (Bar Harbor, ME). Mice were lightly anaesthetized with ketamine and xylazine and infected by the i.n. route (10^4^ c.f.u. in 4 × 5 μl aliquots) or the s.c. route (injection of 50 c.f.u. in 100 μl with a 28 gauge needle at the left inner thigh) with *Y. pestis* strains diluted in PBS. The inoculating dose was confirmed by plating on BHI agar following incubation at 26 °C for 2 days. Mice were monitored twice daily for the length of the experiment. Mice were euthanized by i.p. injection of pentobarbital sodium. All animal infections were performed at least twice and the data combined.

To determine bacterial burden, mice were inoculated i.n. or s.c. with *Y. pestis* strains and euthanized at various times post infection. For i.n. infections, the lungs and spleens were removed, and for s.c. infections, the left inguinal lymph nodes and spleens were removed. Organs were subsequently weighed, homogenized in sterile PBS, serially diluted and plated onto BHI agar. Following incubation at 26 °C for 2–3 days, the c.f.u. per organ was enumerated. To determine survival of mice following infection, mice were inoculated i.n. with *Y. pestis* and derivatives and monitored every 12 h for up to 10 days. Statistical significance was calculated by the Kaplan–Meier test for survival experiments and a two-tailed Mann–Whitney *U*-test for bacterial burden measurements.

### Histopathology

Mice were inoculated i.n with *Y. pestis* strains, and at 48 h post infection mice were euthanized and lungs inflated with 1 ml of 10% neutral buffered formalin via tracheal cannulation. Lungs were removed, fixed in 10% formalin and subsequently embedded in paraffin. Two 5-μm sections, 200 μm apart per lung, were stained with haematoxylin/eosin (H&E) for examination. Tissue embedding, sectioning and staining with haematoxylin/eosin were performed by the Northwestern University Mouse Histology and Phenotyping Laboratory. Slides were imaged using a Zeiss Axioskop/Nuance Camera.

### Innate immune cell quantification

Mice were infected i.n. with *Y. pestis* strains or mock-infected with PBS, and at 48 h post infection mice were euthanized and BAL was performed^38^ using 1 ml PBS for each lavage for a total of five lavages per animal. Samples were centrifuged at 300*g* for 10 min to separate cells and cell debris; the supernatant from the first wash was saved for cytokine analyses (see below). Immune cells contained in the BAL fluid were collected by pooling the pellets from each wash. Cells were resuspended in fluorescence-activated cell sorting (FACS) buffer (2% foetal bovine serum in PBS). A 50-μl aliquot was taken, the total number of cells in BAL fluid were counted on a hemocytometer, and viability was determined by Typran Blue exclusion. Before antibody staining, cells were incubated with Fixable Viability Stain 450 (BD Biosciences) for 15 min at room temperature. Cells were then washed with FACS buffer+0.1% sodium azide and incubated with anti-CD16/32 FcBlock antibody (eBioscience, clone 93) to minimize non-specific binding for 10 min at 4 °C. For *ex vivo* cell surface marker detection, cells were stained with antibodies for CD45 (BD Biosciences, clone 30-F11), CD11b (BD Biosciences, clone M1/70), F4/80 (eBioscience, clone BM8), CD11c (BD Biosciences, clone HL3) and Ly6G (BD Biosciences, clone 1A8). All antibodies were used at 1:100 dilutions in FACS buffer, while Live/Dead cell stain was used at 1:1,000. Cells were washed with FACS buffer and fixed with 2% paraformaldehyde. Before analysis, samples were plated for sterility onto BHI. Samples were analysed using a BD FACSCanto II flow cytometer and FlowJo software. Statistical significance was calculated by the Student's *t*-test.

### Cytokine analysis

At 48 h post inoculation with PBS or the indicated *Y. pestis* strains, the levels of tumour-necrosis factor, interferon-γ, monocyte chemoattractant protein 1 (MCP-1), and interleukin-6 were quantitatively established from the supernatants of collected BAL fluid (see above) using the cytometric bead array technique (BD Cytometric Bead Array Mouse Inflammation Kit, BD Biosciences) as specified by the manufacturer^38^. Before analysis, supernatants were passed through a 0.22-μm filter and plated for sterilization. Data were analysed using the BD Cytometric Bead Array Software. Statistical significance was calculated by the Student's *t*-test.

### Statistics

In all cases, statistical means are graphed and error bars represent s.e.m. Sample sizes (*n*) given represent biological replicates and were not predetermined. For animal experiments, sample sizes were limited by vivarium and ABSL-3 facility capabilities and experimental processing times. Any variation in animal age was matched between groups of interest for comparison and animals were euthanized for processing in the same order as infection. Within a given group, the orders of animal infection and euthanasia were random. The blinding of animal experimental groups was not possible. Student's two-tailed unpaired *t*-test was performed for all instances, unless otherwise indicated. All experiments were analysed with the statistical tests indicated using GraphPad Prism 5. *P* values are indicated as *P≤0.05, **P≤0.01 and ***P≤0.001.

## Additional information

**How to cite this article:** Zimbler, D. L. *et al.* Early emergence of *Yersinia pestis* as a severe respiratory pathogen. *Nat. Commun.* 6:7487 doi: 10.1038/ncomms8487 (2015).

## Supplementary Material

Supplementary Information Supplementary Figures 1-6, Supplementary Tables 1-3 and Supplementary References
