# Supplementary material for: Early emergence of Yersinia pestis as a severe respiratory pathogen
Source: Nat Commun. 2015 Jun 30;6:7487. doi: 10.1038/ncomms8487 (PMC4491175; doi:10.1038/ncomms8487)
Supplement: Supplementary Information — Supplementary Figures 1-6, Supplementary Tables 1-3 and Supplementary References [file ncomms8487-s1.pdf]

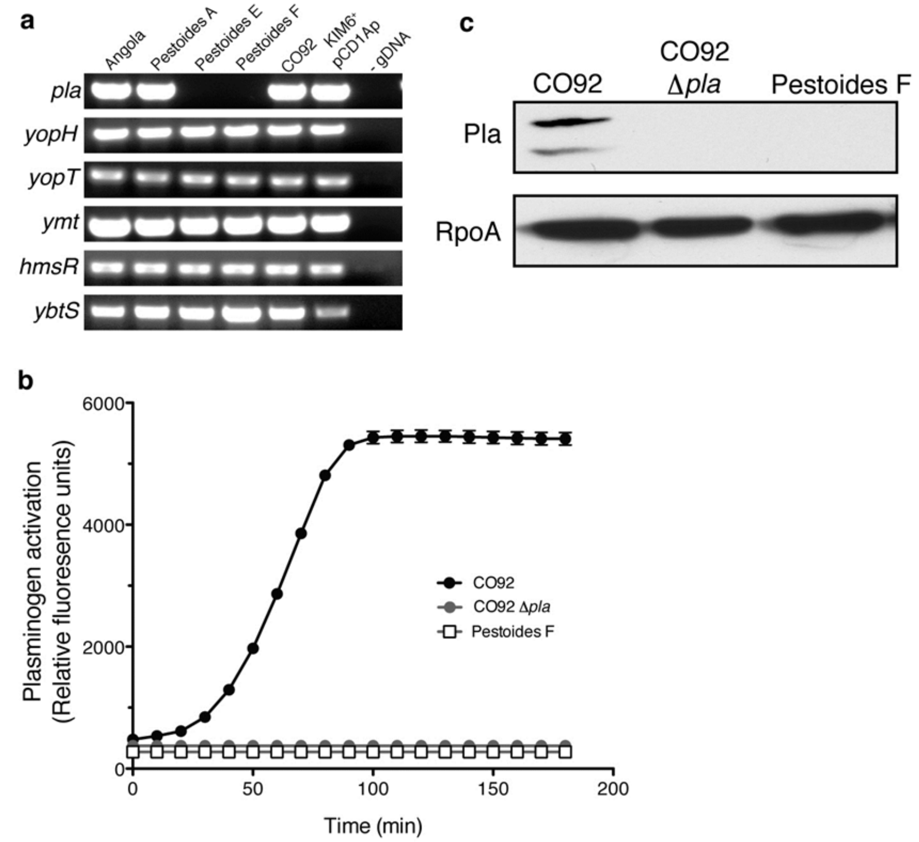

Supplementary Figure 1. Pestoides F does not produce Pla or activate plasminogen (Plg). (a) PCR analysis with primers against known *Y. pestis* virulence loci using gDNA isolated from the indicated *Y. pestis* strains. Data are representative of 2 biological replicates. Full gels are shown in Supplementary Fig 6. (b) Immunoblot analysis of whole-cell lysates from *Y. pestis* CO92, Δ*pla* CO92, or Pestoides F strains cultured at 37˚C with antibodies against Pla and RpoA (as a loading control). Data are representative of 3 biological replicates. Full blots are shown in Supplementary Fig. 6. (c) The Plg-activating ability of *Y. pestis* CO92, CO92 Δ*pla*, and Pestoides F strains cultured at 37˚C. Bacteria were incubated with purified human glu-plasminogen and a fluorescent substrate of plasmin. Data are representative of 3 independent experiments performed in triplicate; error bars represent the s.e.m.

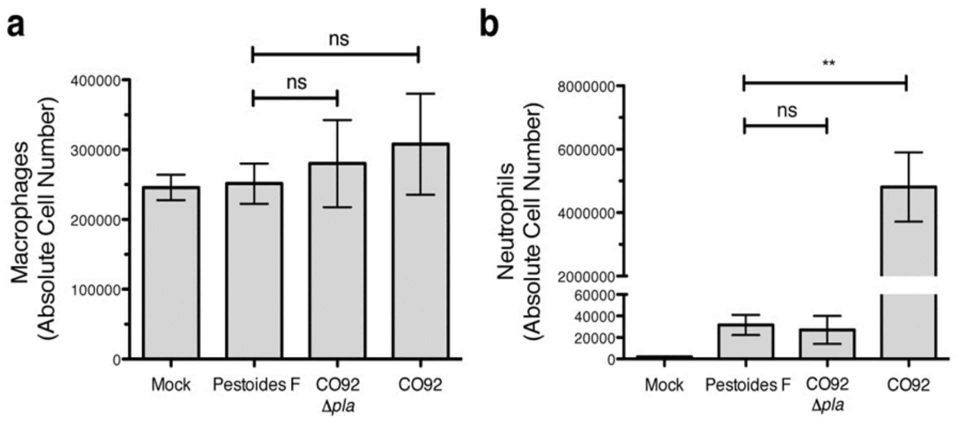

Supplementary Figure 2. Pestoides F is unable to induce a severe inflammatory pneumonia during respiratory infection and is similar to CO92 Δ*pla*. Total number of macrophages ($CD45^{+}F4/80^{+}$) (a) and neutrophils ($CD45^{+}Ly6G^{+}$) (b) collected from BAL fluid at 48 h post-inoculation with PBS (mock) or the indicated *Y. pestis* strains. Data are combined from 2 independent experiments (n=10 for each group); error bars represent the s.e.m. (Student's *t*-test, $^{**}P \leq 0.01$, ns = not significant).

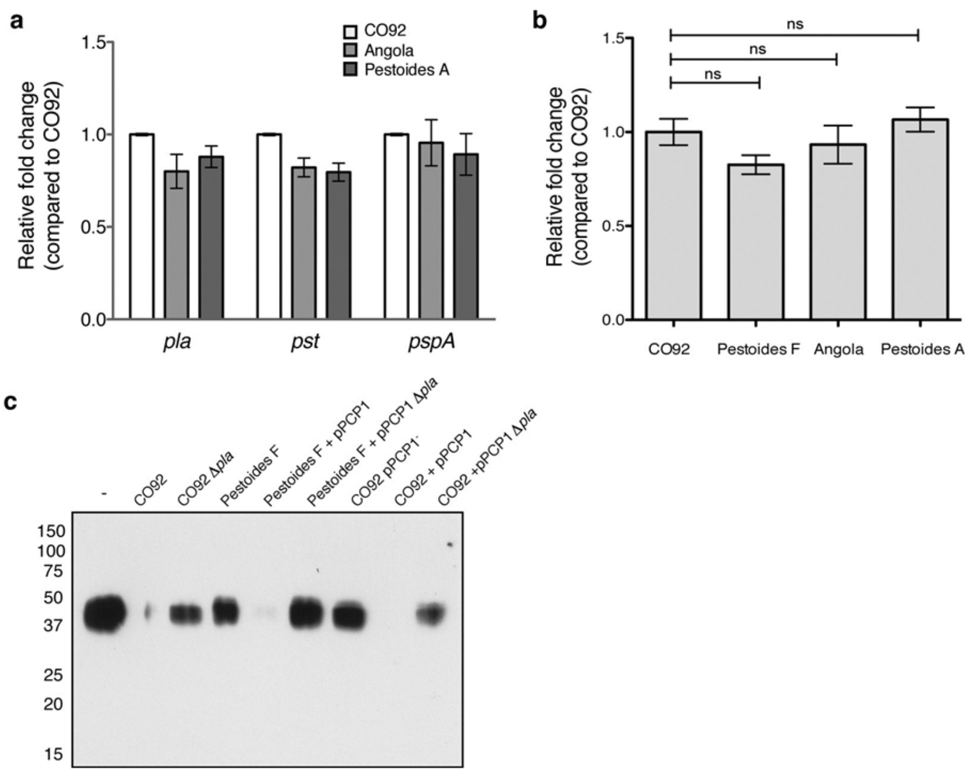

Supplementary Figure 3. Pestoides F is competent to carry pPCP1 and produce active Pla. (a) Relative copy number of pPCP1 (represented by the pPCP1-encoded genes *pla* and *pst*) in *Y. pestis* Angola and Pestoides A compared to CO92 (set at 1). *pspA* was used as a control for a chromosomal gene. Relative copy number for each gene was measured by qPCR from gDNA isolated from cultures grown overnight at 37˚C and normalized to *gyrB*. Data are combined from 3 independent biological replicates repeated twice; error bars represent the s.e.m. (b) *Y. pestis* strains with the P_{pla}-*gfp* reporter construct integrated onto the chromosome were cultured at 37˚C for 6 h, and fold-change in fluorescence compared with *Y. pestis* CO92 (set at 1), normalized to the optical density of the cultures, was determined. Data are combined from 3 independent experiments (*n*=9 for each group); error bars represent the s.e.m. (c) Immunoblot analysis of recombinant mouse FasL following 1 h incubation with the indicated *Y. pestis* CO92 or Pestoides F strains. Numbers to the left indicate molecular weight in kDa. Data are representative of 3 independent experiments. (Student's *t*-test, ns = not significant).

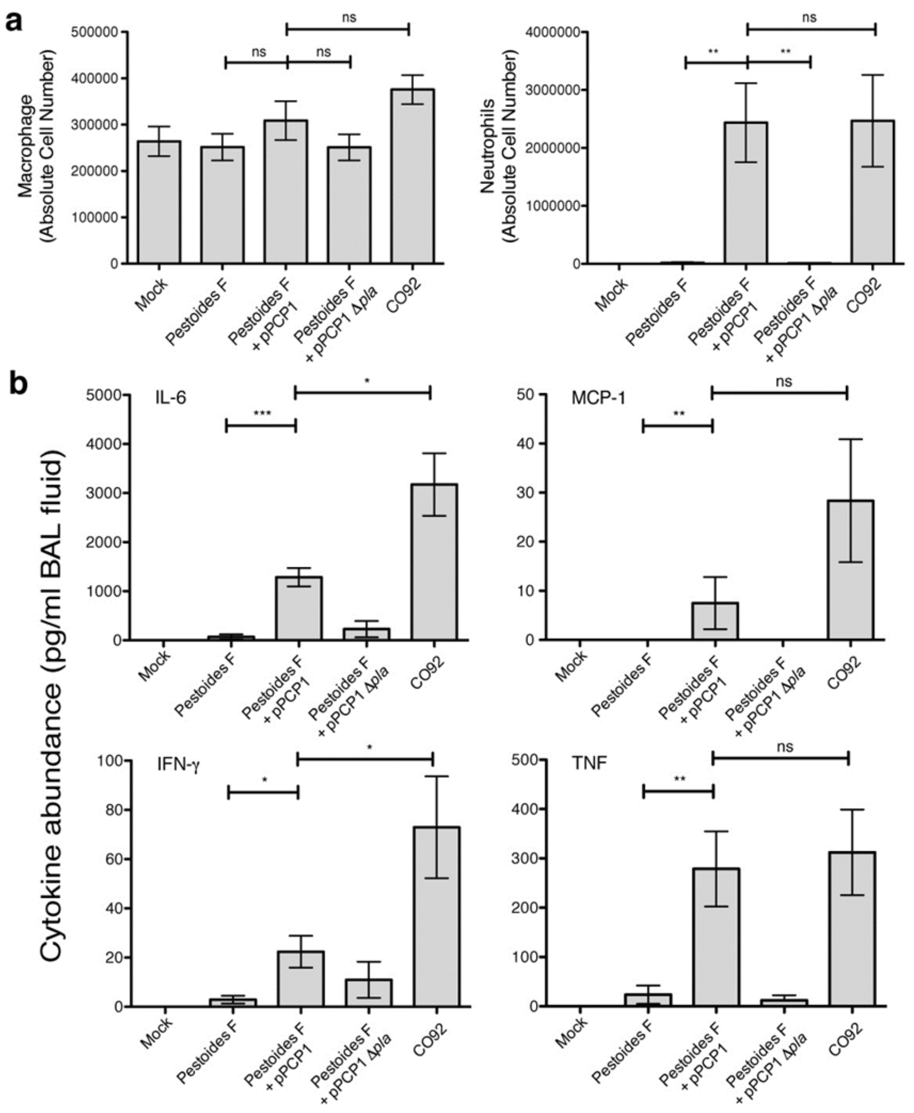

Supplementary Figure 4. **The acquisition of Pla by Pestoides F is sufficient to induce a severe inflammatory pneumonia.** (a) Total number of macrophages ($CD45^{+}F4/80^{+}$) (left) and neutrophils ($CD45^{+}Ly6G^{+}$) (right) collected from BAL fluid at 48 h post-inoculation with PBS (mock) or the indicated *Y. pestis* strains. (b) Abundance of the indicated inflammatory cytokines present in BAL fluid at 48 h post-inoculation. Data are combined from 2 independent experiments (n=10 for each group); error bars represent the s.e.m. (Student's t-test, $*P \leq 0.05$, $**P \leq 0.01$, $*** P \leq 0.001$, ns = not significant).

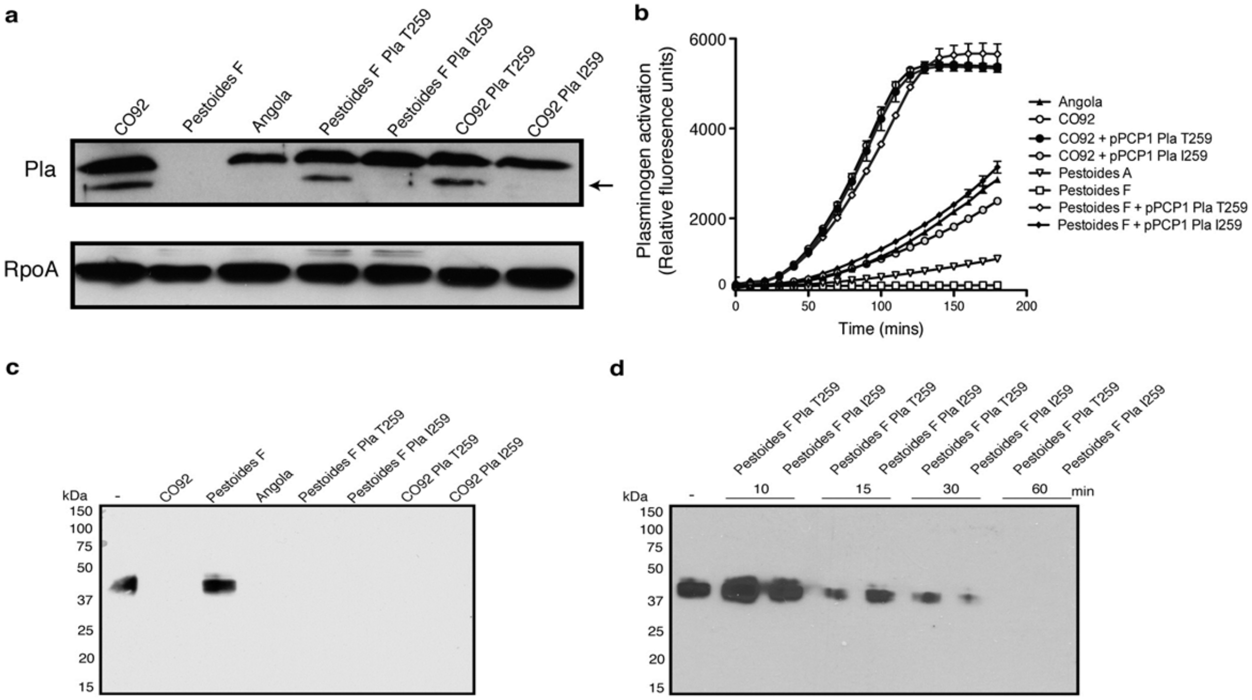

Supplementary Figure 5. The T259 modification of Pla is altered in its interactions with specific substrates. (a) Immunoblot analysis of whole-cell lysates of indicated *Y. pestis* strains cultured at 37ºC with antibodies against Pla and RpoA (as a loading control). The arrow to the right of the Pla blot indicates the autoprocessed from of the T259 Pla variant. Data are representative of 3 independent experiments. Full blots are shown in Supplementary Fig. 6. (b) The Plg-activating ability of the indicated *Y. pestis* CO92 or Pestoides F strains containing either the T259 or I259 variant of Pla, cultured at 37˚C, is shown. Data are representative of 3 independent experiments performed in triplicate; error bars represent the s.e.m. (c) Immunoblot analysis of recombinant mouse FasL following 60 min incubation with the indicated *Y. pestis* CO92 or Pestoides F strains containing either the T259 or I259 variants of Pla. Numbers to the left of the blot indicate molecular weight in kDa. (d) Immunoblot analysis of recombinant mouse FasL following 10, 15, 30 or 60 min incubation with *Y. pestis* Pestoides F containing either the

T259 or I259 variants of Pla. Numbers to the left of the blot indicate molecular weight in kDa. Data are representative of 3 independent experiments.

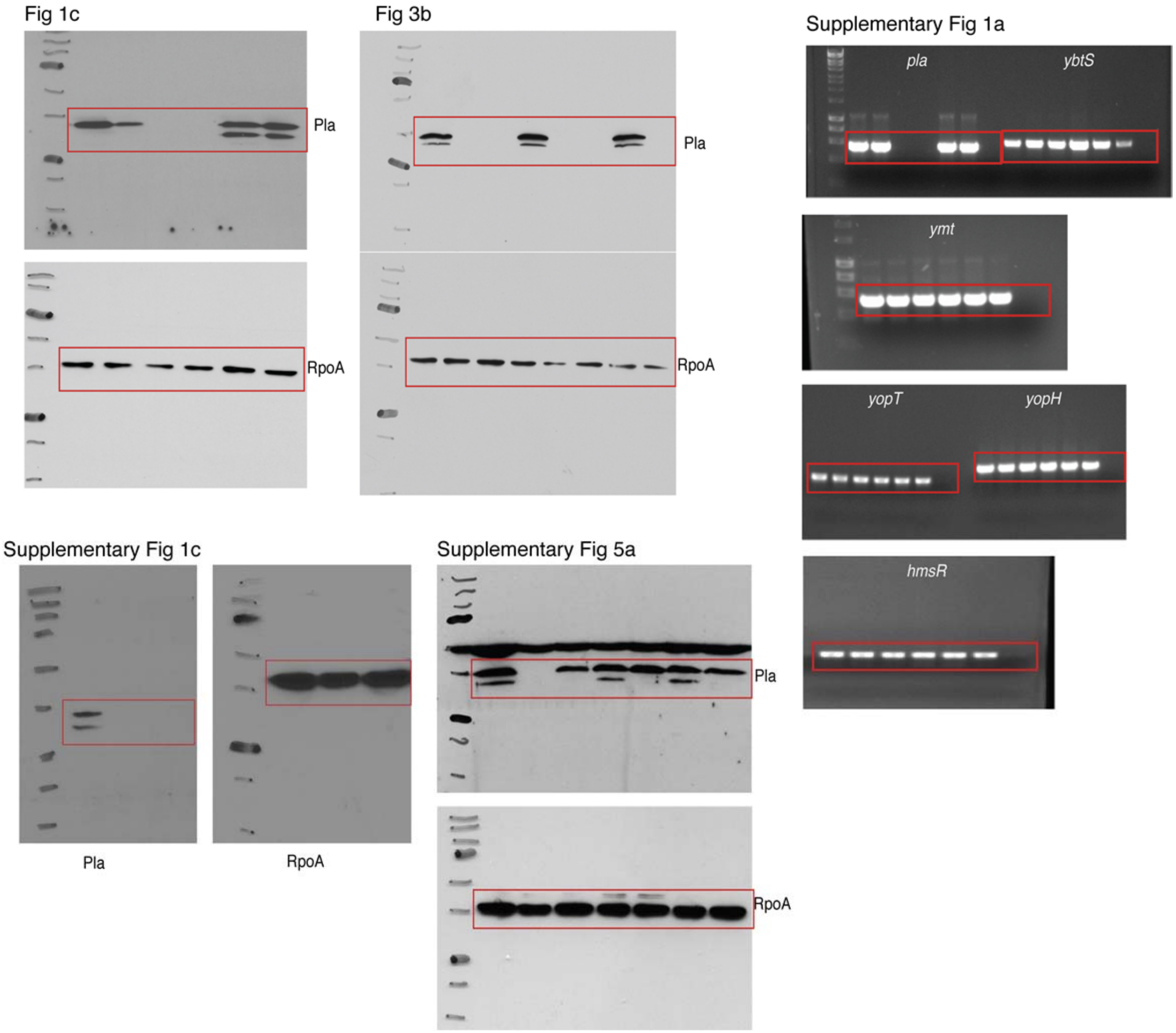

Supplementary Figure 6. Full images of the immunoblots and gels. Boxes highlight regions used in the figures.

Supplementary Table 1. Bacterial strains used in this study

Strains	Designation	Relevant characteristics	Source/reference
Y. pestis			
Angola	SAN189	pCD1$^+$, pMT1$^+$, pPCP1$^+$, pgm$^+$	[1]
Angola LCR$^-$	PAN811	pCD1$^-$, pMT1$^+$, pPCP1$^+$, pgm$^+$	This work
Angola LCR$^-$	PAN837	PAN811 + promoterless gfp	This work
Angola LCR$^-$	PAN838	PAN811 + P*pla*-gfp reporter	This work
CO92	SAN2	pCD1$^+$, pMT1$^+$, pPCP1$^+$, pgm$^+$	[2]; Lab stock
CO92 Δ*pla*	SAN6	Δ*pla*; pCD1$^+$, pMT1$^+$, pPCP1$^+$, pgm$^+$	[3]
CO92	SAN16	pCD1$^+$, pMT1$^+$, pPCP1$^-$, pgm$^+$	Lab stock
CO92	SAN177	SAN16 + pPCP1$^+$	This work
CO92 Δ*pla*	SAN185	Δ*pla*; pCD1$^+$, pMT1$^+$, pPCP1$^+$, pgm$^+$	This work
CO92	SAN205	SAN16 + *pla* I259 on pPCP1	This work
CO92 LCR$^-$	PAN259	pCD1$^-$, pMT1$^+$, pPCP1$^+$, pgm$^+$	[3]
CO92 LCR$^-$ Δ*pla*	PAN314	Δ*pla*; pCD1$^-$, pMT1$^+$, pPCP1$^+$, pgm$^+$	[3]
CO92 LCR$^-$	PAN484	PAN259 + promoterless gfp	[4]
CO92 LCR$^-$	PAN607	PAN259 + P*pla*-gfp reporter	[4]
CO92 LCR$^-$	PAN764	pCD1$^-$, pMT1$^+$, pPCP1$^-$, pgm$^+$	This work
CO92 LCR$^-$	PAN766	PAN764 + pPCP1	This work
CO92 LCR$^-$Δ*pla*	PAN786	PAN766; Δ*pla*	This work
CO92 LCR$^-$	PAN821	PAN766 + *pla* I259 on pPCP1	This work
KIM6$^+$	PAN15	pCD1$^-$, pMT1$^+$, pPCP1$^+$, pgm$^+$	[5]
KIM6$^+$ (pCD1Ap)	SAN187	pCD1Ap$^+$, pMT1$^+$, pPCP1$^+$, pgm$^+$	[5]; this work
Pestoides A	SAN191	pCD1$^+$, pMT1$^+$, pPCP1$^+$, pgm$^+$	[6]
Pestoides A LCR$^-$	PAN812	pCD1$^-$, pMT1$^+$, pPCP1$^+$, pgm$^+$	This work
Pestoides A LCR$^-$	PAN839	PAN812 + P*pla*-gfp reporter	This work
Pestoides A LCR$^-$	PAN840	PAN812 + promoterless gfp	This work
Pestoides E	SAN199	pCD1$^+$, pMT1$^+$, pPCP1$^-$, pgm$^+$	[6]
Pestoides F	SAN87	pCD1$^+$, pMT1$^+$, pPCP1$^-$, pgm$^+$	[6]
Pestoides F	SAN175	pCD1$^+$, pMT1$^+$, pPCP1$^+$, pgm$^+$	This work
Pestoides F Δ*pla*	SAN203	Δ*pla*; pCD1$^+$, pMT1$^+$, pPCP1$^+$, pgm$^+$	This work
Pestoides F	SAN207	SAN87 + *pla* I259 on pPCP1	This work
Pestoides F LCR$^-$	PAN457	pCD1$^-$, pMT1$^+$, pPCP1$^-$, pgm$^+$	This work
Pestoides F LCR$^-$	PAN763	PAN457 + pPCP1	This work
Pestoides F LCR$^-$ Δ*pla*	PAN785	PAN763; Δ*pla*	This work
Pestoides F LCR$^-$	PAN822	PAN763 + *pla* I259 on pPCP1	This work
Pestoides F LCR$^-$	PAN835	PAN457 + P*pla*-gfp reporter	This work
Pestoides F LCR$^-$	PAN836	PAN457 + promoterless gfp	This work
E. coli			
DH5α		Used for DNA recombinant methods	Lab stock
HB101		Used for DNA recombinant methods	Lab stock
S17 lambda pir		Used for DNA recombinant methods	Lab stock

Supplementary Table 2. Plasmids used in this study

Plasmid	Relevant characteristics*	Reference
pPCP1::*kan*	pPCP1 from CO92 with *kan* in the intergenic region downstream from the IS100 ATP-binding protein; Km^R	7
pCD1Ap	pCD1 from KIM with *bla* inserted into *yadA*; Ap^R	8
pKD13	Source of kan^R cassette flanked by FRT sites; Km^R	9
pLB30	Promoterless gfp; Km^R, Ap^R	4
pLB38	P*pla*-gfp reporter; Km^R, Ap^R	4
pTNS2	Carries the Tn7 transposition function; Ap^R	10
pWL204	Carries the lambda red recombination genes; Ap^R	3
pSkippy	Carries FLP recombinase genes; Ap^R	11

*Ap^R, ampicillin resistance; Km^R, kanamycin resistance

Supplementary Table 3. Primers used in this study

Gene	Nucleotide sequence 5' – 3'
yopH 5' 1360	GCAGCAAGAGAGCGGTGATTG
yopH 3'670	GGCATTAAGATCGGCGCGTAC
yopT 5' 290	GGCGTGGGCTGACATTTGGC
yopT 3' 777	CTCGTGTGCCCGCGGTGAGA
ymt 5'1	ATCACTGAAGTACTGCGGAATTCGC
ymt 3' 532	GGGAGCCATAAGCAGAAGAACC
hmsR 3'1300	TTACCCAACGCGCACGCTTAC
hmsR 5' 800	ACTGGTCGGTATTCTTCGAACCC
ybtS 5' 209	AATGGGCGGTGGATTCCATT
ybtS 3' 810	CGCCTTATTATGCTCCGGT
pla 5' 397	GACCTCAATGTGAAAGGCTGGTTACACC
pla 3' 498	ACCACCTGTAGCTGTCCAACTGAAAC
pst 5' 139	CAGGCAGATCCACATAATGTGG
pst 3' 20	GAATGGCAGATCCACATAATGTGG
gyrB.r.3'	ATTGGTAAAGGTCTGGAAACTTGGCC
gyrB.f.5'	TCGCCGTGAAGGTAAAGTTC
pspA 5' 318	TGAAGTGGCAACCGTGGACGAAA
pspA 3'422	AAGGTCAATGCCTGTTGTCTGGCT
pla 5'- 500	CGCCTGCTGGCTGCACTTGTCGTTG
P4 *pla* 5' 938	GGTCGACGGATCCCCGGAATGAAAAATACAGATCATATCTCTC TTTTCATC
pla 3' + 500	CTGGAGAGCAAGTAATGAGAACATTA
P1 *pla* 3'- 3	GAAGCAGCTCCAGCCTACACCATTAGACACCCTTAATCTCTCTG CATGAAC
pla 5' T259I	GGAGGTACTCAGATCATTGATAAGAAT
pla 3' T259I	ATTCTTATCAATGATCTGAGTACCTCC
pla 3' + 127	GCGCCCCGTCATTATGGTGAAAAAG
P1 *pla* 3' 939	GAAGCAGCTCCAGCCTACACCTCAGAAGCGATATTGCAGACCC GCC

Supplementary References

1 Eppinger, M. *et al.* Genome sequence of the deep-rooted *Yersinia pestis* strain Angola reveals new insights into the evolution and pangenome of the plague bacterium. *J Bacteriol* **192**, 1685-1699 (2010).

2 Parkhill, J. *et al.* Genome sequence of *Yersinia pestis*, the causative agent of plague. *Nature* **413**, 523-527 (2001).

3 Lathem, W. W., Price, P. A., Miller, V. L. & Goldman, W. E. A plasminogen-activating protease specifically controls the development of primary pneumonic plague. *Science* **315**, 509-513 (2007).

4 Bellows, L. E., Koestler, B. J., Karaba, S. M., Waters, C. M. & Lathem, W. W. Hfq-dependent, co-ordinate control of cyclic diguanylate synthesis and catabolism in the plague pathogen *Yersinia pestis*. *Mol Microbiol* **86**, 661-674 (2012).

5 Fetherston, J. D., Schuetze, P. & Perry, R. D. Loss of the pigmentation phenotype in *Yersinia pestis* is due to the spontaneous deletion of 102 kb of chromosomal DNA which is flanked by a repetitive element. *Mol Microbiol* **6**, 2693-2704 (1992).

6 Achtman, M. *et al.* Microevolution and history of the plague bacillus, *Yersinia pestis*. *Proc Natl Acad Sci U S A* **101**, 17837-17842 (2004).

7 Forman, S. *et al.* *yad*BC of *Yersinia pestis*, a new virulence determinant for bubonic plague. *Infect Immun* **76**, 578-587 (2008).

8 Gong, S., Bearden, S. W., Geoffroy, V. A., Fetherston, J. D. & Perry, R. D. Characterization of the *Yersinia pestis* Yfu ABC inorganic iron transport system. *Infect Immun* **69**, 2829-2837 (2001).

9 Datsenko, K. A. & Wanner, B. L. One-step inactivation of chromosomal genes in Escherichia coli K-12 using PCR products. *Proc Natl Acad Sci U S A* **97**, 6640-6645 (2000).

10 Choi, K. H. *et al.* A Tn7-based broad-range bacterial cloning and expression system. *Nature methods* **2**, 443-448 (2005).

11 Price, P. A., Jin, J. & Goldman, W. E. Pulmonary infection by *Yersinia pestis* rapidly establishes a permissive environment for microbial proliferation. *Proc Natl Acad Sci U S A* **109**, 3083-3088 (2012).
